# Demonstration and Pantomime in the Evolution of Teaching

**DOI:** 10.3389/fpsyg.2017.00415

**Published:** 2017-03-22

**Authors:** Peter Gärdenfors

**Affiliations:** ^1^Cognitive Science, Lund UniversityLund, Sweden; ^2^Stellenbosch Institute for Advanced Studies, Wallenberg Research CentreStellenbosch, South Africa

**Keywords:** demonstration, evolution of language, gesture, mental simulation, mimesis, pantomime, teaching

## Abstract

Donald proposes that early *Homo* evolved mimesis as a new form of cognition. This article investigates the mimesis hypothesis in relation to the evolution of teaching. The fundamental capacities that distinguish hominin teaching from that of other animals are demonstration and pantomime. A conceptual analysis of the instructional and communicative functions of demonstration and pantomime is presented. Archaeological evidence that demonstration was used for transmitting the Oldowan technology is summarized. It is argued that pantomime develops out of demonstration so that the primary objective of pantomime is that the onlooker learns the motoric patterns shown in the pantomime. The communicative use of pantomime is judged to be secondary. This use of pantomime is also contrasted with other forms of gestures. A key feature of the analysis is that the meaning of a pantomime is characterized by the force patterns of the movements. These force patterns form the core of a model of the cognitive mechanism behind pantomime. Finally, the role of pantomime in the evolution of language is also discussed.

## Introduction

Donald (1991, 2001) formulates a ‘mimesis hypothesis’, which states that a specific form of cognition (and a corresponding culture) mediated between that of the ancestor we have in common with the apes and that of modern humans. In brief, Donald proposes that while ape culture is based on associational learning, early *Homo* evolved a new form of cognition. The basis for this was that the body could be used volitionally to do what somebody else is doing (imitation), to represent external events for the purpose of communication (mime, gesture) and to rehearse a given skill by matching performance to a goal.

Donald (2012) expands the mimesis hypothesis and emphasizes that a key feature of the human memory system is our ability to voluntarily retrieve a particular memory. He notes: “Nonhuman animals can learn skills with appropriate conditioning, but their performance can be retrieved only by external cues that elicit conditioned responses. Voluntary recall, as in self-triggered conscious retrieval, the kind of recall needed to practice a skill, is absent” (Donald, 2012, p. 275). The ability to initiate the internal cuing process that triggers a memory of a previous performance he calls ‘autocuing’. His thesis can help explain why one finds evidence of apprenticeship among the hominins, but not in the apes or other non-human animals (Sterelny, 2012).

Humans do not only engage in rehearsal, they also teach extensively. In contrast, there is very restricted evidence concerning intentional teaching in non-human animals. A central question is: How did intentional teaching evolve along the hominin line? This article focuses on the role of mimesis in that process. A starting point is the analysis of teaching in Gärdenfors and Högberg (2017) where five levels of intentional teaching are distinguished: (1) evaluative feedback (approval and disapproval), (2) drawing attention, (3) demonstrating (showing how to do), (4) communicating concepts (teaching categories), and (5) explaining relationships between concepts. The analysis is summarized in Section “Evolutionary Levels of Teaching”. A crucial step separating humans from other animals is ‘showing how to do’.

I will expand on this analysis by providing an analysis of two forms of mimesis involved in showing how to do, namely, *demonstration* and *pantomime* (see Demonstration and Pantomime). Archaeological evidence for early uses of demonstration will also be presented briefly. Pantomime will then be put in contrast with other forms of gestures in Section “Relations between Pantomime and Gesture”. I argue that the meaning of a pantomime is characterized by the force patterns of the movements. In Section “The Cognitive Mechanism of Pantomime”, a model of the cognitive mechanism behind pantomime is presented. I suggest that the human mind adds representations of forces when planning and imagining actions. “Enactment and the Evolution of Communication” then discusses the role of pantomime in the evolution of language.

## Evolutionary Levels of Teaching

### Imitation and Emulation

Individuals, human or non-human, can learn without being taught. An individual can learn by him/herself, but often the learning takes place in a social context. In social learning, the learning individual observes the behavior of a knowledgeable individual (the model), while the model does not adapt its behavior to make it easier for the first individual to learn (Nielsen et al., 2012). An example is the nut-cracking behavior of chimpanzees (Boesch and Boesch, 1982; Biro et al., 2006). It can take up to four years for adolescent chimpanzees to learn from adults and become proficient at cracking open palm nuts with stone hammers and anvils. Adults rarely help to correct hammering techniques or encourage the young.

Tomasello (1999) distinguishes between learning by *emulation*, where the learner observes the outcomes of the model’s actions and tries to reach the same outcome (goal-oriented learning), and learning by *imitation*, where the learner observes the sequence of the model’s actions and tries to perform the same actions (process-oriented learning) (see also Zentall, 2001; Tehrani and Riede, 2008).

The ‘artificial fruit’ experiments by Whiten et al. (2005) have been designed to investigate the differences between emulation and imitation. Early results indicated that chimpanzees emulate while children imitate, but later studies suggest that the situation is more complicated (Horowitz, 2003; Whiten et al., 2009). Imitating the use of familiar motor actions in novel situations also seem to be easier for chimpanzees than copying new motor actions (Myowa-Yamakoshi and Matsuzawa, 1999). Froese and Leavens (2014) argue that, in many situations, chimpanzees as well as children directly *perceive* the intention of the model and that they emulate in such situations. Children, and to some extent chimpanzees (Buttelmann et al., 2007) imitate when an action is constrained by conventions (or just arbitrarily made up) so that the intention of the model cannot be perceived. Froese and Leavens (2014) predict that the more an action relies on conventions, the more faithfully it will be imitated.

The apprenticeship culture that evolved among hominins (Sterelny, 2012), presumes a well-established ability to rehearse. But even though rehearsal is best seen as a form of self-imitation – I remember how I performed a sequence of actions last time and what the outcome was, now I can try to improve on my previous performance – it is cognitively different from imitation. Self-imitation depends on autocuing of memories (Donald, 2012).

### Levels of Intentional Teaching

Learning by imitation and emulation does normally not involve any form of teaching intention on the part of the model. In contrast, demonstration and pantomime are intentional forms of teaching. I next present a summary of the five levels of intentional teaching in Gärdenfors and Högberg (2017) (for another classification of teaching, see Kline, 2015).

#### (1) Evaluative Feedback

The teacher approves or disapproves of the learner’s behavior (Castro and Toro, 2004). For example, if the learner is about to eat a poisonous plant, the teacher may show disapproval of this and thereby the learner will hopefully learn to avoid that kind of food. Animal data on this form of teaching include chimpanzee mothers taking away dangerous food from infants, and gorilla, chimpanzee and macaque mothers facilitating and encouraging infants’ independent locomotion (Maestripieri, 1995, 1996; Whiten, 1999).

#### (2) Drawing Attention

Here, the teacher’s intention is that the learner focuses on a particular object, action or feature. Among humans, drawing attention is often achieved by pointing (but other methods are also used). Non-human animals draw attention in particular via alarm calls. However, in most cases these signals seem to be non-intentional and not dependent on what the conspecifics know and do not know (although see Crockford et al., 2012). Several bird species, including hens of domestic fowl, peck at the ground and call chickens to draw attention to suitable food (Nicol and Pope, 1996).

#### (3) Demonstrating

This involves intentionally showing somebody else how to perform a task or how to solve a problem. Demonstrating builds on advanced mindreading both for the teacher and for the learner. It presumes that the teacher understands the lack of knowledge in the learner and that the learner experiences that there is something to learn. This kind of teaching also requires that the teacher and the learner *jointly attend* to the demonstration.

#### (4) Communicating Concepts

For example, teaching categorizations of plants or animals is an important form of transmission of knowledge. In modern human societies, the main method to teach a concept is to use a word (or gestural sign) standing for the concept together with pointing or some other technique for drawing the attention to what falls under the category. Concept teaching relies on mindreading since it presumes that the learner understands that the teacher is intentionally using a gesture or a sound as a *communicative sign*, that is, that the gesture or sound is used to ‘stand for’ something else (Zlatev et al., 2005a) (see Enactment and the Evolution of Communication).

#### (5) Explaining Relationships between Concepts

The teacher’s intention in explaining is typically that the learner understands the causal relationship between two concepts. For example, if this arrow is dipped in poison, then it will kill an animal that it hits, and wet wood is not good for lighting a fire. Unlike the previous ones, this level presumes that the teacher uses a symbolic language (spoken or gestured) that can refer to things that are not present in the teaching situation.

For all five levels, it is assumed that the teacher has an intention that the learner learns something that s/he would not learn without the intervention of the teacher. Gärdenfors and Högberg (2017) analyze the requirements concerning mindreading (theory of mind) for each of the five levels. As one goes up the levels, the requirements on communication capacities also increase. In particular, level 5 requires symbolic communication, while indexical or iconic gesturing may be sufficient for levels 2–4 (see Relations between Pantomime and Gesture).

Most of the teaching of non-human animals is non-intentional, but there are cases of intentional teaching on levels (1) and (2). With one possible exception (see below), it is only humans who have been shown to teach according to levels (3–5). This leads to the central question of why only hominins have the capacity to demonstrate, that is, to reach level (3). This capacity seems to have generated a breakthrough in hominin teaching and in transmission of culture. Before the question can be approached, a conceptual analysis of ‘showing how to do’ is necessary. In the following section, this capacity will be divided into two related forms of enactment, namely, demonstration and pantomime.

## Demonstration and Pantomime

### The Structure of Demonstration

Demonstrating involves intentionally showing somebody else how to perform a task or to solve a problem. Demonstration is a central element in ‘natural pedagogy’ and seems to be present in all human societies (Csibra and Gergely, 2009, 2011). Showing a child how a toy functions, how to brush her teeth or how to tie her shoelaces are well-known everyday examples. Gärdenfors and Högberg (2017) argue that demonstration was already used in teaching the 2.5 million year old Oldowan stone knapping technology (see Archaeological Evidence for Demonstration below) and that learning this technology does not presume any symbolic form of communication (see Enactment and the Evolution of Communication).

When a teacher demonstrates to a learner how to perform a certain task, the following criteria are characteristic:

(D1) The demonstrator *actually performs* the actions involved in the task.
(D2) The demonstrator makes sure that the learner *attends* to the series of actions.
(D3) The demonstrator’s *intention* is that the learner can perceive the right actions in the correct sequence.
(D4) The demonstrator *exaggerates* and slows down some of the actions in order to facilitate for the learner to perceive important features.

When the learner tries to imitate the demonstrated action, the teacher reacts with approval or disapproval (level 1). A demonstration may or may not be accompanied with verbal comments. There is typically some form of *feedback*, maybe just a nod of the head, from the learner, indicating that he or she has understood what is being demonstrated.

In criterion (D1), it is presupposed that the performance is voluntary, that is, autocued in the sense of Donald (2012). As regards criterion (D2), Csibra and Gergely (2009, p. 149) point out that “human communication is often preceded, or accompanied, by ostensive signals that (i) disambiguate that the subsequent action (for example, a tool-use demonstration) is intended to be communicative and (ii) specify the addressee to whom the communication is addressed”. Gergely et al. (2007) provide experimental evidence for the importance of the ostensive nature of the teacher’s behavior.

Criteria (D2) and (D3) entail that demonstrating builds on advanced mindreading both for the teacher and for the learner. The most efficient (and the typical) way to satisfy (D2) is that the teacher and the learner achieve *joint attention*, but other means of making the learner attend are also possible. As mentioned in the previous section, (D3) presumes that the teacher understands the lack of knowledge in the learner and that the learner experiences that there is something to learn.

It should be noted that demonstration is not confined to direct teaching but can also be used in other situations. One pertinent example is that after training, an apprentice can demonstrate to a teacher or bystanders that she has learned to perform a particular task. Another example is as part of certain forms of narrative, say in a court case, where a witness demonstrates how somebody behaved (the border line between this and pantomime may be vague in this case).

As a comment on (D4), it should be noted that demonstration presumes that the learner will learn by imitation rather than by emulation. It not only the goal of the demonstration that is important but the sequence of actions leading to it. Highlighting initial and final states of an action helps the learner to segment the sequence of actions as well as the preconditions for the initiation of the action and the properties of its final result.

When demonstrating in front of children, adults exaggerate their movements, they emphasize the beginning and end of movements and they pause before and after the task. In addition to establishing joint intention, this is also part of the ostensive signals that a teacher uses to control the attention of the learner (see Gergely et al., 2007). This form of demonstration has been called *Motionese* (Brand et al., 2002; Rohlfing et al., 2004). In this way, children are assisted in processing and interpreting complex actions.

A few observations of chimpanzees showing somebody else how to perform an action have been reported: a chimp mother can show her infant how to hold a stone in order to crack a nut against an anvil stone (Boesch, 1991). I do not consider this to be a good example of demonstration, since the mother only helps the infant hold the stone correctly, but she does not show how to hit the nut. Consequently, this behavior is perhaps better seen as facilitating (which is classified as a non-intentional form of teaching by Gärdenfors and Högberg, 2017; see also Kline, 2015).

A possible example of demonstration in non-human animals is presented by Bates et al. (2010). African female elephants occasionally simulate oestrus when pregnant or lactating. This behavior occurs when a young inexperienced female behaves inappropriately in relation to the bulls. The older females’ simulated oestrus makes it possible for them to show how the dominant bull should be approached. The fact that the simulated behavior is targeted only to young females in need of help possibly qualifies it as demonstration. The behavior is rare, however, and the evidence is not strong so it is problematic to determine to what extent the adults understand the situation of the young female and thereby whether conditions (D2)–(D4) are fulfilled.

Even if apes do not demonstrate themselves, they can be taught to understand demonstration. In so-called do-as-I-do experiments, the subjects (apes or children) are shown actions of different kinds and they are then either asked to “do the same thing” (if the verbal command is learned) or (if not) the spontaneous handling of the object is recorded (Bering et al., 2000; Bjorklund et al., 2000; Bjorklund and Bering, 2003). Mother-reared chimpanzees seem to do less well while enculturated apes can outperform human children on certain tasks (Tomasello et al., 1993). An explanation for this difference could be that the mother-reared chimpanzees have been less exposed to conventional and symbolic actions. The intentions behind such actions are opaque and therefore emulation is impossible (Froese and Leavens, 2014).

### The Structure of Pantomime

The second form of enactment involved in teaching is pantomime. I view pantomime as a special case – perhaps the most central – of the mimetic ability that Donald (1991, 2001, 2012) has identified as a crucial step in human evolution.

Pantomime may have several functions (see Relations between Pantomime and Gesture), but here I focus on pantomiming for somebody how to perform a certain task. The following criteria are characteristic of this function (see Arbib, 2012, pp. 218–219) for a related analysis):

(P1) The mimer performs the *movements* of the actions in the task without actually performing the actions.
(P2) The mimer makes sure that the learner *attends* to the series of actions.
(P3) The mimer’s *intention* is that the learner can perceive the right actions in the correct sequence.
(P4) The mimer *exaggerates* and slows down some of the actions in order to facilitate for the learner to perceive important features.

Criteria (P2)–(P4) are the same criteria as (D2)–(D4) and the same comments as in the previous subsection apply to them. The crucial difference is in criterion (P1), since in pantomime the actual actions are not performed, but some more or less simplified version of them. Again, (P1) presumes that the pantomime is voluntary (autocued). As regards, (P3), Arbib (2012, pp. 217–218) writes: “Where imitation is the generic attempt to reproduce movements performed by another, whether to master a skill or simply as part of a social interaction, pantomime is performed with the intention of getting the observer to think of a specific action or event.” Another difference with respect to demonstration is that pantomime is *displaced* in the sense of Hockett (1960), that is, it can refer to entities not present in the immediate environment (Zywiczynski et al., 2016, section 3.8). For example, I can pantomime how to open a difficult door as part of describing for you how to get into my apartment that you are borrowing tomorrow.

It should be noted that there are degrees of pantomime. For example, a tennis teacher can pantomime a special swing using a racket (but not hitting a ball) or she can pantomime the swing by just using her arm. Another example is that a boy can pantomime a frog jumping by using his full body or by just using his hand. When pantomiming with the hand, the body parts of the frog are typically not represented but only the overall pattern of the frog’s movement. Some researchers restrict pantomime to enactment involving the whole body (e.g., Gullberg, 1998, p. 97)), but I take a broader perspective and allow that only parts of the body are used in a pantomime.

Many researchers see pantomiming as a form of gesturing. In Section “Relations between Pantomime and Gesture”, I compare pantomiming to other types of gestures. Pantomime can be used for other purposes than teaching, for example for narrating or as part of telling a joke. I will argue that pantomime is primarily not used in communicative gestures. [In line with this thesis, Zywiczynski et al. (2016) distinguish between cognitive and communicative forms of pantomime.] Apart from teaching, pantomime can, for example, be used in autocued rehearsal (think of a boxer in front of a mirror) of the form discussed by Donald (2012).

From an evolutionary perspective, an interesting question is whether non-human animals, in particular apes, can pantomime. Language-trained apes seem to be able to pantomime (see below in relation to pretense). Researchers are divided on whether other apes (wild or in captivity) have the capacity. On the one hand, Russon and Andrews (2010, p. 316) have collected evidence for pantomiming in orangutans. They conclude that “pantomime could have been within the grasp of the common human-great ape ancestor”. However, most of the evidence they analyze conforms to the observation by Gibson (2013, p. 209) that apes only gesture about requested actions (typically play or sex) of the addressee. This means that what is referred to by the gesturer is the behavior of the addressee and not some other object (see e.g., Boesch and Tomasello, 1998). [This analysis fits well with Reddy’s (2005) account of the development of sociality, which starts with mutual attention where the attention is focused on the other and only later develops into joint attention directed to a third object.] On the other hand, some researchers are more skeptical, for example Zuberbühler (2013, p. 136), who claims about apes that “pantomiming is conspicuously absent, apart from isolated anecdotes”.

Pantomime is a form of *pretense*. When you pretend, you use *two* representations of the same object or action – your perception of the object or action and an imagined version of it (Leslie, 1987). For example, when a girl pretends that a shoe is a telephone, she knows that it is a shoe but she simultaneously ‘sees’ it as a telephone that she can talk into. By *suppressing* her perception she can use her imagination instead (see The Cognitive Mechanism of Pantomime). Her image is a deliberately false representation of the world. In accordance with this, Leslie (1987) maintains that such imagined events are necessary to be able to pretend. In the example above, the perception of the shoe must be suppressed and the performance completed with knowledge about telephones and how they are used that the girl accesses from her memory. Leslie writes that small children’s pretense play “is an early symptom of the human mind’s ability to characterize and manipulate its own attitudes to information. [……] In short, pretense is an early manifestation of what has been called a *theory of mind*” (Leslie, 1987, p. 416).

Clark writes about ‘depictions’ that he defines as “physical scenes that people stage for others to use in imagining the scenes depicted” (Clark, 2016, p. 325). It is clear that demonstration and pantomime fall under this definition. In line with Leslie’s argument, he writes that “depictions are the core of children’s make-believe play” (Clark, 2016, p. 324) and he argues that a depiction builds on a ‘double-reality principle’ since it “has two realities: its base, or raw execution; and its appearance, the features that are intended to be depictive” (Clark, 2016, p. 327).

There are some examples of pretense play that have been recorded for chimpanzees and gorillas that have grown up among humans. The bonobo Kanzi often pretends that a make-believe dog or gorilla is biting him, or that he is pursuing and biting someone (Savage-Rumbaugh et al., 1998, p. 60). When he was young, the chimpanzee Austin often pretended to be eating, sometimes even with make-believe plate and make-believe spoon. The second example is a clear case of pantomime.

In contrast, demonstration does not presume the double worlds that are required for pantomime. Pantomime can be seen as a combination of demonstration and pretense. This suggests that demonstration puts less demand on the cognitive capacities of the demonstrator. Hence, from both an evolutionary and a developmental viewpoint it is reasonable that, in teaching contexts, demonstration should appear earlier than pantomime.

### Archaeological Evidence for Demonstration

Taking an archaeological perspective, a question is what is the earliest period of hominin history where indications of enactment capacities can be found. A partial answer to this question is proposed by Gärdenfors and Högberg (2017), who argue that already the transmission of how to manufacture Oldowan tools requires demonstration. If this argument is correct, demonstration has been used by hominins for at least 2.5 million years.

The argument builds on the capacity to master *core maintenance*, which is achieved by detaching flakes from the core in a way that makes it possible to strike further flakes from it later. Experimental studies have shown that core maintenance requires planning. To learn the technique, a teacher must demonstrate a setup that allows a flake to be detached in a way that facilitates the detachment of another flake, which in turn facilitates for the next flake to be detached, etc. To achieve this the teacher must demonstrate (or pantomime) an appropriate way to hold the core and the correct angle and movement of the arm and hand holding a hammer stone when detaching a flake. Then the learner must practice, typically for a long time, to master the technique.

Some researchers have claimed that the behavior of Oldowan tool-producing hominins is also achievable by apes (Wynn et al., 2011). Their main supporting evidence for the claim is the knapping behavior of the bonobos Kanzi and Panbanisha, both trained to knap by human knappers. However, Toth et al. (1993) show that Kanzi did not achieve the skill level of Oldowan knappers. The bonobos never voluntarily rehearsed knapping as it had been demonstrated to them. Donald’s (2012) thesis concerning the apes’ lack of voluntary retrieval of memories entails that they are unable to rehearse. Kanzi only engaged in the kind of knapping demonstrated to him when encouraged by his teachers, or when the reward box was loaded (Toth et al., 1993). In particular, no signs of core maintenance are visible in Kanzi’s knapping.

## Relations Between Pantomime and Gesture

### Pantomime as a Form of Gesture

There are many different attempts to define what characterizes gestures, some of which are very general (e.g., Armstrong et al., 1995). Most of the definitions are not based on an evolutionary perspective (although see Brinck, 2001 for an analysis of the origins of pointing). Kendon’s (2004, p. 15) definition is restricted to ‘utterance uses’ of gestures, that is, gesture used in a communicative function, often together with speech. Pantomime, however, also has uses that are not connected to utterances, so a more comprehensive definition is appropriate. Following, Zlatev et al. (2005a, p. 23), I therefore consider as gestures “goal-directed communicative body movements, i.e., such that require interpretation from an audience for achieving the gesturer’s goal”. Here I only consider *representational* gestures, so that, for example, beat and emblem gestures are excluded (Kendon, 2004, Chaps. 9–11). [In beat gestures, the hand is used to mark the rhythm of the speech. Emblems, such as thumbs up and the V-formed peace sign, are conventional gestures.] Among representational gestures, a basic distinction is between *indexical* gestures, where the ground is one of spatio-temporal contiguity (e.g., pointing) and *iconic* gestures, where the ground is of similarity (e.g., pantomime).

McNeill (2013, p. 483) describes pantomime as gesture without speech. However, vocal sounds can be parts of a pantomime. For example, I can pantomime an up-and-down movement by changes in the pitch of my voice or imitate the sound of an animal while pantomiming its movements. Furthermore, this characterization brings out a tension in the origin of pantomime. McNeill and other gesture researchers describe pantomime in terms of communication, while my position if that the primary function of pantomime is non-communicative.

This distinction also shows up when determining what is the *intention* of a pantomime. There seem to be two different types: Firstly, I can pantomime an *action* that I want you to *copy*. This is the typical case in a teaching situation. Secondly, I can pantomime an action as part of a *message* (request, command, warning, narrative, etc.). In the gesture literature, the second use of pantomime (communicative act) seems to have been in focus and it is this meaning that is used when pantomime as an art is referred to. Here, I am mainly interested in the first use. According to the proposed definition, a demonstration is also a gesture, but it is only used for the first type of intention. However, since pantomime can also be used for the second type, it has a broader use than demonstration.

Even though the evidence for pantomiming apes is weak, they are capable of producing other forms of gestures. Non-human gestures are typically *dyadic*, where only two individuals are involved, but no external object [although see Pika and Bugnyar (2011) on referential gestures in ravens]; for example when an ape gestures where it wants to be groomed or to show which copulation position it desires (Tanner and Byrne, 1996; Zlatev et al., 2005a; Pollick and de Waal, 2007). An example of a *triadic* gesture is a human pointing to an object in the presence of another individual in order to achieve joint attention (see The Cognitive Mechanism of Pantomime).

### Semantic Domains of Gestures

McNeill (1992) distinguishes between *character viewpoint gestures*, where the one who gestures enacts the movements of the object (e.g., showing how a fish was trying to swim away when it was on your hook), and *observer viewpoint gestures*, where the one who gestures relates to the object from the outside (e.g., showing how big the fish was by enacting touching its head and tail) (see also Gullberg, 1998; Parrill, 2009). Gestures for object properties are typically *observer viewpoint gestures*, while gestures for actions are typically *character viewpoint gestures.* Pantomime clearly belongs to character viewpoint gestures.

A characteristic aspect of pantomimes is that they express actions. This point can be clarified by considering the *semantic domains* of different types of gestures. In Gärdenfors (2014), I argue that for adjectives, verbs and prepositions, the meaning of a single word only depends on a single semantic domain. For example, ‘red’ refers to a region of color space, ‘push’ to a region of vectors in force space, and ‘near’ to a region of physical space.

A version of the hypothesis can also be extended to the semantics of gestures. There exist three types of representational gestures corresponding to three different types of semantic domains:

(i) *Location*. This involves the domain of physical space, which is the characteristic referential domain for pointing gestures.
(ii) *Object Properties*. Gestures can represent the shape, size, length, height, depth and maybe other properties of an object. These properties each belong to an object category domain (Gärdenfors, 2014, Ch. 6).
(iii) *Actions*. According to the analysis presented in previous work (Gärdenfors, 2007, 2014, Chap. 8; Gärdenfors and Warglien, 2012), actions can be represented as *patterns of forces.* The underlying semantic domain for this kind of gesture is thus the force domain.

The importance of the domain analysis is that a pantomime can now be characterized as a gesture that for its semantic function *principally involves the force domain*. If I am pantomiming the jumping of a frog, my hand or my full body will exhibit some typical force pattern of a frog’s movement. In this way, the represented action is iconically enacted. This analysis accords with Kendon’s (2004, p. 160), since he identifies pantomime with enactment that is oriented toward actions.

The domain analysis presented here is complicated by the fact that a pantomime can be combined with information about the properties of an object that is part of the depicted event. For example, a gesture showing how a glass was put on a table can be a combination of a pantomime of the placing movement and a hand-shape that indicates the shape of the object that is placed. Gullberg (2011) has investigated language-specific gestures for placement events. For a language, such as English and French, that has only one main placement verb (‘put’ and ‘mettre’), a native speaker only gestures the movement but not the shape of the object that is being placed. In contrast, for a language, such as Dutch and Swedish, that has several placement verbs (corresponding to ‘set’, ‘stand’, and ‘lay’) that indicate the shape of the object moved, a native speaker gestures both the movement and the shape of the object.

Clark’s (2016) analysis of *depiction* has close similarities to gestures. The examples he presents all seem to fall under the property and action domains. He writes (ibid.) that “depicting things is different from locating things”, which makes it clear that the location domain is not included in depictions. (However, bimanual gestures where one hand describes the location of an object and the other the movement of the object are not uncommon.) His distinction between ‘prop’ and ‘actor’ depictions (Clark’s 2016, p. 331) corresponds to my distinction between gestures for objects properties and gestures for actions (see Clark, 2016, p. 336) for the different kinds of ‘prop’ depictions). Therefore, my analysis of pantomime seems closely related to Clark’s notion of actor depictions although he does not consider the evolutionary roots of depiction.

## The Cognitive Mechanism of Pantomime

### Mental Representation

My objective in this section is to outline how the mechanism behind pantomime can be derived from more fundamental cognitive capacities. First of all, it should be recalled that animals, including humans, *represent* the world around them in different ways. Following Humphrey (1993) and Gärdenfors (2003), the different forms of mental representation can be divided into three kinds.

(i) *Sensations* that are the immediate sensory impressions.

Our subjective world of experiences is based on sensations: tastes, smells, colors, itches, pains, sensations of cold, sounds, etc. (what philosophers of mind call qualia). They provide an awareness of the world.

(ii) *Perceptions* that are interpreted sensory impressions.

The brain is full of mechanisms that contribute new information to the sensory input. In particular, there are many well-studied examples concerning the visual process. For example, an object is perceived to have *contours* – they are part of the information that the visual process *constructs* in order to generate perceptions. The brain interprets the sensation and constructs objects that stand out from a continuous visual influx.

(iii) *Imaginations* (or images) that are not directly generated by sensory impressions.

Being able to use imaginations requires that one can *suppress* the sensations one has for the moment; otherwise they will come into conflict with the representation. Evoking a memory of an event is a typical example of an imagination. Glenberg (1997) says that imaginations put reality in quarantine. This form of suppression is the basic mechanism behind pretense and autocuing.

### Planning Actions and Pantomime

Forming a plan involves representing different actions, that is, different approaches to reaching a goal (Gulz, 1991). Jeannerod (1994) argues that “actions are driven by an internally represented goal rather than directly by the external world”. By exploiting its capacity to imagine, the agent can *simulate* a number of different actions in order to ‘see’ their consequences and to evaluate them (Gärdenfors, 2003; Grush, 2004). After such simulations, the agent can choose the most appropriate action to perform.

Hostetter and Alibali (2008) present their ‘gesture-as-simulated-action’ framework as a mechanism to explain how representational gestures emerge from perceptual and motor simulations. They write that “an action generator is responsible for planning the form of a gesture and this generator accesses visuospatial images that are active in working memory” (Hostetter and Alibali, 2008, p. 507). The mechanism I propose here is compatible with their framework, but since I am mainly concerned with pantomime, I focus on motor simulation and want to explain how the action generator functions.

An important property of a simulator is that it does not need to rely exclusively on the signals coming from sense organs: it can also *add on* new types of information that can be useful in simulating (Gärdenfors, 2004; Grush, 2004). It does not matter much if the added information has no direct counterpart in the surrounding world as long as the simulations produce the right result, that is, lead to appropriate actions.

In particular, I submit that different kinds of simulators produce variables that are used in *causal reasoning*. As has been shown by Povinelli (2000) and others, monkeys and apes are surprisingly restricted in their reasoning about physical causes of phenomena. On the other hand, even very small human children show strong signs of interpreting the world with the aid of hidden forces and other causal variables. Gopnik (1998, p. 104) claims that “other animals primarily understand causality in terms of the effects of their own actions on the world. In contrast, human beings combine that understanding with a view that equates the causal power of their own actions and those of objects independent of them”. Apparently, humans have more advanced causal simulators than other animals (see also Gärdenfors, 2003, section 2.8, and Woodward, 2011). The forces involved are primarily physical, but can be metaphorically extended to ‘mental’ forces involved in threats, persuasions, etc.

As I have discussed in previous sections, there seems to be very limited, if any, evidence that other animals can demonstrate or pantomime. My explanation for this is that only humans use the force patterns of actions in their mental simulations and are therefore able to represent actions via gestures. From this it follows that pantomime involves character viewpoint gestures. My explanation builds on the hypothesis that the human brain, but not that of other species, adds forces as hidden variables in its simulations of actions and their consequences (Runesson, 1994; Gärdenfors and Warglien, 2012). This explains why other species do not pantomime.

A consequence of this thesis is that if you can show someone else the relevant forces involved in an action, then this may be sufficient for the addressee to understand which action you are representing. Understanding the intention of a pantomime is, however, cognitively more demanding than understanding a demonstration. The meaning of a demonstration is clear as soon as the addressee understands that it is performed in a teaching context. For a pantomime, the addressee must also understand that the teacher intends the pantomime to *stand for* a real action and that the teacher intends the addressee to realize this. In the following section this will be called the ‘communicative sign function’.

The mechanism I propose for pantomime is thus that when you want to show an action to someone – either for the purpose of teaching the other individual how to perform the action or as a part of a communicative act – you mentally extract the relevant force patterns and perform them using your body. The upshot is that if this mechanism works, then pantomime is sufficient to communicate actions. A pantomime can therefore be seen as a caricature of a demonstration. As mentioned earlier, the pantomime can also be complemented with some props such as the tools involved in the represented action. In contrast to a real action (or a demonstration), the goal of a pantomime is not to achieve a real result in the world, but to make the addressee grasp the appropriate forces involved in the action.

## Enactment and the Evolution of Communication

### The Mimesis Hierarchy

In this article my focus is on the use of demonstration and pantomime in teaching and the evolutionary importance of these methods. The analysis has, however, strong connections to the evolution of communicative systems. Following Zlatev et al. (2005a), I use the following criteria for distinguishing different acts (communicative or of another type).

#### Cross-modality

The act involves a cross-modal mapping between proprioception (kinesthetic experience) and exteroception (normally dominated by vision).

This condition expresses that mimesis involves the body, including specific parts such as the hand and the vocal tract. According to the motor theory of speech perception (Liberman and Mattingly, 1985) speech likewise involves such a cross-modal mapping.

#### Volition

The act is realized by bodily motion that is, or can be, under conscious control.

The condition expresses what Donald (2012) calls autocuing with respect to bodily movements.

#### Representation

The motions involved in the act correspond to – either iconically or indexically – some action, object or event, but at the same time are differentiated from it by the agent.

As discussed in Section “The Structure of Pantomime”, having access to the double world of pretense (Leslie, 1987) and depiction (Clark, 2016) is necessary for representation.

#### Communicative Sign Function

The agent intends for the act to stand for some action, object or event for an addressee, and for the addressee to realize this.

This criterion is related to Grice’s (1957) criterion of meaning, but it is weaker since it only involves second order intention (the agent intends that the addressee understand the communicative intentions), but not third order (Gärdenfors, 2003, section 6.3; Bar-On, 2013).

#### Symbolicity

The act is fully conventional, that is, a part of mutual knowledge, and breaks up into meaningful sub-acts that relate systematically to each other and to other similar acts.

On the basis of these criteria, Zlatev et al. (2005a) define a ‘mimesis hierarchy’ that is summarized in **Table 1**.

**Table 1:** The mimesis hierarchy.

Stage	Definition	Examples
Proto-mimesis	A bodily act involving *cross-modality* with proprioception, but lacking *volition* or *representation* (or both)	Facial expressions, bodily synchronization
Dyadic mimesis	An interpersonal or intrapersonal bodily act displaying *volition* and *representation*, but not *communicative sign function*	Shared attention, imperative pointing, mirror self-recognition, do-as-I-do imitation
Triadic mimesis	As dyadic mimesis but also involving *communicative sign function*	Joint attention, declarative pointing, pantomime
Post-mimesis	As triadic mimesis, but also involving *symbolicity*	Sign language

*Definitions of the four evolutionary stages and examples of corresponding types of acts.*

It should be noted that demonstrations do not exhibit the communicative sign function, but they involve an intention that the addressee imitates what is demonstrated. Hence demonstration falls somewhere between dyadic and triadic mimesis. Of the three examples of triadic mimesis in **Table 1**, joint attention and declarative pointing are analyzed in Zlatev et al. (2005a), but not pantomime. According to the mimesis hierarchy, pantomime is triadic since it is cross-modal, volitional, representational and it has a communicative sign function. However, it is not conventional, even though a repeated pantomime can quickly become a convention within a community. In contrast, demonstrations never become conventions.

Apes reach dyadic mimesis, but hardly triadic (joint attention is contested, see Leavens and Racine, 2009). In contrast, triadic mimesis in the forms of joint attention, declarative pointing and pantomime appear early in the behavior of human children. As regards intentional teaching, Strauss et al. (2002) show that three-year-olds can teach by demonstration and that five-year-olds can teach by explaining rules (levels 3 and 5, respectively, in the analysis of Gärdenfors and Högberg (2017)). Together with the criteria in **Table 1**, these observations suggest that triadic mimesis, in form of the ability to demonstrate and pantomime and the ability to engage in joint attention, is an early evolved component of the human cognitive repertoire that distinguishes us from that of other animals.

Triadic mimesis involves two key functions of mindreading (theory of mind): Joint attention and understanding the intentions of others (for the communicative sign function). It is therefore likely that human mindreading capacity has been a major driving force for the evolution of triadic mimesis (Zlatev et al., 2005a,b). Since triadic mimesis is necessary for advanced forms of cooperation (Brinck and Gärdenfors, 2003; Gärdenfors et al., 2012) its role as a major step in the evolution of human cognition is emphasized.

### The Role of Pantomime in the Evolution of Language

In my analysis, I have brought forth two main functions for pantomime: The first is an invitation to copy – the teaching function. The second is the communication function. I submit that the teaching function is the more primitive. The following quotation from McNeill (2013, section 5.3) supports this position: “Natural gesture signals in modern apes have an incipient action quality as well, the characteristic of which is that an action is cut short and the action-stub becomes a signifier; a kind of metonymy. The slow-to-emerge precursor from 5 million years ago to 2 million years ago may have built up a gesture language that derived from instrumental actions as envisioned in gesture-first. It would have been an evolution track leading to pantomime.” McNeill thus sees the teaching function as the more primitive and the communicative functions as a metonymical extension. This accords with my position that pantomime is primarily a non-communicative mechanism.

**FIGURE 1:**
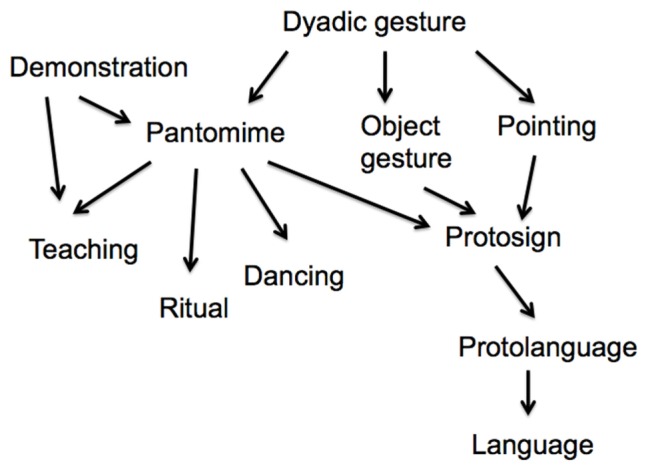
**The position of pantomime in the evolution of hominin cognition**.

Returning to the communicative use of pantomime, I have already noted that pantomime is displaced so that it can be used to communicate about absent or future events. However, pantomime is not conventional or symbolic. Nevertheless, pantomime is a useful tool for planning cooperative actions (Gärdenfors, 2013). Pantomime has been argued to be a precursor to protosign and protolanguage (see **Figure 1**). Arbib (2012, pp. 219–226) suggests that protosign develops by conventionalization out of pantomime and other gestures. He writes that “[p]antomime is not itself part of protosign but rather a scaffolding for creating it” (Arbib, 2012, p. 224). However, in its function as an invitation to copy, pantomime is also a probable precursor to *dance* and *ritual*. These evolutionary paths, which I will not follow here, further strengthen the centrality of pantomime in the evolution of the human mind (see **Figure 1**).

Given my partitioning of gesture references into locations, object properties and actions, these three categories of gestures can be seen as protodemonstratives, protoadjectives, and protoverbs. Gestures for nouns would typically develop out of characteristic properties, but they may also emerge out of verbs.

My characterization of pantomime has been rather narrowly confined to depicting actions. One may take a more comprehensive perspective and take pantomime to encompass the combination of gestures for actions with gestures for object properties (or objects) and locations. Such an interpretation seems to be intended by Zywiczynski et al. (2016, section 3.7), who write that “pantomimic acts are ‘the size of’ propositions or utterances rather than smaller component units; rather than being elements of a larger communicative whole, they express complete, *self-contained* communicative acts”. This proposal is consistent with Arbib’s (2012) idea that early communication consisted of holophrases (see also Zlatev et al., unpublished).

On this broader account, pantomimes refer to *events*. According to a cognitive semantic analysis of events (Gärdenfors and Warglien, 2012; Warglien et al., 2012; Gärdenfors, 2014), events consist of an *action*, normally performed by an *agent*, that affects a *patient* and leads to a certain *result*. This theory builds on conceptual spaces (Gärdenfors, 2000, 2014) where actions are modeled as force vectors (or patterns) and results as vectors describing change in some property of the patient. My hypothesis is that in later stages of the evolution of communication, the holophrases represented by a pantomime are broken down into smaller semantic elements representing the components of the event, that is, agent, patient, action and result (see Gärdenfors, 2014).

The way mothers talk to their children is called ‘Motherese’ or ‘child-directed speech’. Distinguishing features are that the pitch of the voice is high and that the stresses are exaggerated (Fernald, 1992). Motherese can be seen as a form of ‘communicative demonstration’ where vowels, prosody and pauses are exaggerated so that the infant with greater ease can pick up the relevant meaning of what is communicated.

It is possible to express movement patterns via prosody. For example, it is common to depict a falling object by a vocal sound (or by music in cartoons) with falling pitch. In this way, pantomime may have played a role in the evolution of spoken language. In addition to a mapping between pitch patterns and the force patterns of actions, Studdert-Kennedy and Goldsmith (2003) argue that phonemes have a gestural origin. Following MacNeilage (1998), they propose that the mammalian capacities for sucking, licking and chewing have been exapted as pantomimed gestures that have then evolved into proto-syllables. To this they add that “[t]he initial impetus for ‘reuse’ of articulators (and so for the emergence of combinatorial mechanisms) would then have come from the simple facts that the articulators were few in number” (Studdert-Kennedy and Goldsmith, 2003, p. 240).

## Conclusion

The starting point of this article is that the crucial step separating human teaching from that of other animals is ‘showing how to do’. To improve our understanding of this evolutionary step, I have provided a conceptual analysis of demonstration and pantomime that are the two main forms of mimesis involved in showing how to do. I have proposed that pantomime develops out of demonstration and that the primary objective of a pantomime therefore is that the onlooker *learns* the motoric pattern shown in the pantomime. Pantomime is often classified among gestures, but I have argued that the communicative function of pantomime is secondary to the instructional one.

My argument expands Donald’s (1991) idea of mimesis as a key factor for the evolution of human cognition. He has recently argued that language co-evolves with culture building on distributed cognitive systems. He writes that there are two preconditions for such systems: “a very general ability to rehearse and refine *skills* (evident early in hominin evolution in tool-making), and the emergence of *material culture* as an external (to the brain) memory record that could retain and accumulate knowledge across generations” (Donald, 2016, p. 1). In accordance with the position taken here, he argues that skills must have evolved before language.

As the complexity of technology and societal practices increased over time, the challenges for new generations to achieve knowledge about the manufacturing and use of tools, food, medicine etc., also increase (see also Csibra and Gergely, 2011, p. 1154). This leads to greater evolutionary benefits of demonstration for transmitting this kind of knowledge to the next generation. An advanced material culture cannot be preserved between generations without teaching. In brief, innovations, of any kind, increase the demands for teaching.

As regards implications of demonstration and pantomime for the evolution of language, an important question is why the hominins (and not other species) had a demand for a symbolic language that acted as a selective force. In earlier publications, I have suggested that language is necessary for the advanced forms of *cooperation* that have evolved along the hominin line, namely, planning for future interaction and indirect reciprocity (Gärdenfors et al., 2012; Gärdenfors, 2013). Teaching should, however, also be seen as a form of cooperation and the later stages in the model of Gärdenfors and Högberg (2017) – communicating concepts and explaining relations between concepts – require advanced forms of communication. It is difficult to say which, if any, of these forms of cooperation has been a dominating force behind the evolution of a symbolic communication system. It is possible that all forms have contributed to the evolution of human cognition and communication. Pantomime is, however, a crucial step in the evolution of any of these forms.

## Author Contributions

The author confirms being the sole contributor of this work and approved it for publication.

## Conflict of Interest Statement

The author declares that the research was conducted in the absence of any commercial or financial relationships that could be construed as a potential conflict of interest.

## References

[B1] Arbib M. (2012). *How the Brain Got Language: The Mirror System Hypothesis.* Oxford: Oxford University Press 10.1093/acprof:osobl/9780199896684.001.0001

[B2] Armstrong D. E. Stokoe W. C. Wilcox S. (1995). *Gesture and the Nature of Language.* Cambridge: Cambridge University Press 10.1017/CBO9780511620911

[B3] Bar-On D. (2013). Origins of meaning: Must we ‘go Gricean’? *Mind Lang.* 28 342–375. 10.1111/mila.12021

[B4] Bates L. A. Handford R. Lee P. C. Njiraini N. Poole J. H. (2010). Why do african elephants (*Loxodonta africana*) simulate oestrus? An analysis of longitudinal data. *PLoS ONE* 5:e10052 10.1371/journal.pone.0010052 PMC2850927 20383331

[B5] Bering J. M. Bjorklund D. F. Ragan P. (2000). Deferred imitation of object-related actions in human-reared juvenile chimpanzees and orangutans. *Dev. Psychobiol.* 36 218–232. 10.1002/(SICI)1098-2302(200004)36:3<218::AID-DEV5>3.0.CO;2-K 10737867

[B6] Biro D. Sousa C. Matsuzawa T. (2006). “Ontogeny and cultural propagation of tool use by wild chimpanzees at Bossou, Guinea: case studies in nut cracking and leaf folding,” in *Cognitive Development in Chimpanzees* eds Matsuzawa T. Tomonaga M. Tanaka M. (Tokyo: Springer) 476–508.

[B7] Bjorklund D. F. Bering J. M. (2003). A note on the development of deferred imitation in enculturated juvenile chimpanzees (*Pan troglodytes*). *Dev. Rev.* 23 389–412. 10.1016/S0273-2297(03)00021-2

[B8] Bjorklund D. F. Bering J. M. Ragan P. (2000). A two-year longitudinal study of deferred imitation of object manipulation in a juvenile chimpanzee (*Pan troglodytes*) and orangutan (*Pongo pygmaeus*). *Dev. Psychobiol.* 37 229–237. 10.1002/1098-2302(2000)37:4<229::AID-DEV3>3.0.CO;2-K 11084604

[B9] Boesch C. (1991). Teaching among wild chimpanzees. *Anim. Behav.* 41 530–532. 10.1016/S0003-3472(05)80857-7

[B10] Boesch C. Boesch H. (1982). Optimisation of nut-cracking with natural hammers by wild chimpanzees. *Behaviour* 83 265–286. 10.1163/156853983X00192

[B11] Boesch C. Tomasello M. (1998). Chimpanzee and human cultures. *Curr. Anthropol.* 39 591–614. 10.1086/204785

[B12] Brand D. A. Baldwin L. A. Ashburn R. J. (2002). Evidence for ‘motionese’: modifications in mothers’ infant-directed action. *Dev. Sci.* 5 72–83. 10.1111/1467-7687.00211

[B13] Brinck I. (2001). Attention and the evolution of intentional communication. *Pragmat. Cogn.* 9 255–272. 10.1075/pc.9.2.05bri

[B14] Brinck I. Gärdenfors P. (2003). Co-operation and communication in apes and humans. *Mind Lang.* 18 484–501. 10.1111/1468-0017.00239

[B15] Buttelmann D. Carpenter M. Call J. Tomasello M. (2007). Encultured chimpanzees imitate rationally. *Dev. Sci.* 10 F31–F38. 17552931 10.1111/j.1467-7687.2007.00630.x

[B16] Castro L. Toro M. A. (2004). Cumulative cultural evolution: the role of teaching. *J. Theor. Biol.* 347 74–83. 10.1016/j.jtbi.2014.01.006 24434740

[B17] Clark H. (2016). Depicting as a method of communication. *Psychol. Rev.* 123 324–347. 10.1037/rev0000026 26855255

[B18] Crockford C. Wittig R. M. Mundry R. Zuberbühler K. (2012). Wild chimpanzees inform ignorant group members of danger. *Curr. Biol.* 22 142–146. 10.1016/j.cub.2011.11.053 22209531

[B19] Csibra G. Gergely G. (2009). Natural pedagogy. *Trends Cogn. Sci.* 13 148–153. 10.1016/j.tics.2009.01.005 19285912

[B20] Csibra G. Gergely G. (2011). Natural pedagogy as evolutionary adaptation. *Philos. Trans. R. Soc. B Biol. Sci.* 366 1149–1157. 10.1098/rstb.2010.0319 PMC3049090 21357237

[B21] Donald M. (1991). *Origins of the Modern Mind: Three Stages in the Evolution of Culture and Cognition.* Cambridge, MA: Harvard University Press.

[B22] Donald M. (2001). *A Mind so Rare: The Evolution of Human Consciousness.* New York, NY: Norton.

[B23] Donald M. (2012). “Evolutionary origins of autobiographical memory: a retrieval hypothesis,” in *Understanding Autobiographical Memory: Theories and Approaches* eds Berntsen D. Rubin D. C. (Cambridge: Cambridge University Press) 269–289. 10.1017/CBO9781139021937.020

[B24] Donald M. (2016). Key cognitive preconditions for the evolution of language. *Psychon. Bull. Rev.* 24 204–208. 10.3758/s13423-016-1102-x 27368636

[B25] Fernald A. (1992). “Meaningful melodies in mothers’ speech to infants,” in *Nonverbal Vocal Communication: Comparative and Developmental Approaches* eds Papousek H. Jürgens U. Papousek M. (Cambridge: Cambridge University Press) 262–282.

[B26] Froese T. Leavens D. A. (2014). The direct perception hypothesis: perceiving the intention of another’s action hinders its precise imitation. *Front. Psychol.* 5:65 10.3389/fpsyg.2014.00065 PMC3927096 24600413

[B27] Gärdenfors P. (2000). *Conceptual Spaces: The Geometry of Thought.* Cambridge, MA: MIT Press.

[B28] Gärdenfors P. (2003). *How Homo Became Sapiens: On the Evolution of Thinking.* Oxford: Oxford University Press.

[B29] Gärdenfors P. (2004). Emulators as sources of hidden variables. *Behav. Brain Sci.* 27 403 10.1017/S0140525X04280098

[B30] Gärdenfors P. (2007). “Evolutionary and developmental aspects of intersubjectivity,” in *Consciousness Transitions: Phylogenetic, Ontogenetic and Physiological Aspects* eds Liljenström H. Århem P. (Amsterdam: Elsevier) 281–305. 10.1016/B978-044452977-0/50013-9

[B31] Gärdenfors P. (2013). “The evolution of semantics: sharing conceptual domains,” in *The Evolutionary Emergence of Language* eds Botha R. Everaert M. (Oxford: Oxford University Press) 139–159.

[B32] Gärdenfors P. (2014). *The Geometry of Meaning: Semantics Based on Conceptual Spaces.* Cambridge, MA: MIT Press.

[B33] Gärdenfors P. Brinck I. Osvath M. (2012). “Coevolution of cooperation, cognition and communication,” in *New Perspectives of the Symbolic Species* eds Stjernfelt F. Deacon T. Schilhab T. (Berlin: Springer) 193–222.

[B34] Gärdenfors P. Högberg A. (2017). The archaeology of teaching and the evolution of Homo docens. To appear in Current Anthropology. 10.1086/691178 [Epub ahead of print].

[B35] Gärdenfors P. Warglien M. (2012). Using conceptual spaces to model actions and events. *J. Semant.* 29 487–519. 10.1093/jos/ffs007

[B36] Gergely G. Egyed K. Király I. (2007). On pedagogy. *Dev. Sci.* 10 139–146. 10.1111/j.1467-7687.2007.00576.x 17181712

[B37] Gibson K. (2013). “Talking about apes, birds, bees, and other living creatures: language evolution in light of comparative animal behavior,” in *Thinking and Speaking in Two Languages* ed. Pavlenko A. (Bristol: Multilingual Matters) 143–169.

[B38] Glenberg A. (1997). What memory is for. *Behav. Brain Sci.* 20 1–19. 10.1017/s0140525x97000010 10096994

[B39] Gopnik A. (1998). Explanation as orgasm. *Minds Mach.* 8 101–118. 10.1023/A:1008290415597

[B40] Grice P. (1957). Meaning. *Philos. Rev.* 66 377–388. 10.2307/2182440

[B41] Grush R. (2004). The emulation theory of representation: motor control, imagery and representation. *Behav. Brain Sci.* 27 377–442. 10.1017/S0140525X04000093 15736871

[B42] Gullberg M. (1998). *Gesture as a Communication Strategy in Second Language Discourse: A Study of Learners of French and Swedish.* Lund: Lund University Press.

[B43] Gullberg M. (2011). “Thinking, speaking and gesturing about motion in more than one language,” in *Thinking and Speaking in Two Languages* ed. Pavlenko A. (Bristol: Multilingual Matters) 143–169.

[B44] Gulz A. (1991). *The Planning of Action as a Cognitive and Biological Phenomenon.* Ph.D. thesis, Lund University Lund.

[B45] Hockett C. F. (1960). The origin of speech. *Sci. Am.* 203 88–96. 10.1038/scientificamerican0960-88 14402211

[B46] Horowitz A. (2003). Do humans ape? Or do apes human? Imitation and intention in humans (*Homo sapiens*) and other animals. *J. Comp. Psychol.* 117 325–336. 10.1037/0735-7036.117.3.325 14498809

[B47] Hostetter A. B. Alibali M. W. (2008). Visible embodiment: gestures as simulated action. *Psychono. Bull. Rev.* 15 495–514. 10.3758/PBR.15.3.495 18567247

[B48] Humphrey N. K. (1993). *A History of the Mind.* London: Vintage Books.

[B49] Jeannerod M. (1994). The representing brain, neural correlates of motor intention and imagery. *Behav. Brain Sci.* 17 187–202. 10.1017/S0140525X00034026

[B50] Kendon A. (2004). *Gesture: Visible Action as Utterance.* Cambridge: Cambridge University Press 10.1017/CBO9780511807572

[B51] Kline M. A. F. (2015). How to learn about teaching: an evolutionary framework for the study of teaching behavior in humans and other animals. *Behav. Brain Sci.* 38 1–17. 10.1017/S0140525X14000090 24856634

[B52] Leavens D. A. Racine T. P. (2009). Joint attention in apes and humans: Are humans unique? *Conscious. Stud.* 16 240–267.

[B53] Leslie A. M. (1987). Pretense and representation: the origins of ’theory of mind’. *Psychol. Rev.* 94 412–426. 10.1037/0033-295X.94.4.412

[B54] Liberman A. M. Mattingly I. G. (1985). The motor theory of speech perception revised. *Cognition* 21 1–36. 10.1016/0010-0277(85)90021-6 4075760

[B55] MacNeilage P. F. (1998). The Frame/Content theory of evolution of speech production. *Behav. Brain Sci.* 21 499–511. 10.1017/S0140525X98001265 10097020

[B56] Maestripieri D. (1995). First steps in the macaque world: Do rhesus mothers encourage their infants’ independent locomotion? *Anim. Behav.* 49 1541–1549. 10.1016/0003-3472(95)90075-6

[B57] Maestripieri D. (1996). Maternal encouragement in nonhuman primates and the question of animal teaching. *Hum. Nat.* 6 361–378. 10.1007/BF02734206 24203124

[B58] McNeill D. (1992). *Hand and Mind: What Gestures Reveal about Thought.* Chicago, IL: University of Chicago Press.

[B59] McNeill D. (2013). “The co-evolution of gesture and speech, and downstream consequences,” in *Body – Language – Communication: An International Handbook on Multimodality in Human Interaction* Vol. 1 eds Müller C. Cienki A. Fricke E. Ladewig S. McNeill D. Tessendorf S. (Berlin: De Gruyter Mouton) 480–512.

[B60] Myowa-Yamakoshi M. Matsuzawa T. (1999). Factors influencing imitation of manipulatory actions in chimpanzees (*Pan troglodytes*). *J. Comp. Psychol.* 113 128–136. 10.1037/0735-7036.113.2.128 10384721

[B61] Nicol C. J. Pope S. J. (1996). The maternal feeding display of domestic hens is sensitive to perceived chick error. *Anim. Behav.* 52 767–774. 10.1006/anbe.1996.0221

[B62] Nielsen M. Subiaul F. Galef B. Zentall T. Whiten A. (2012). Social learning in human and nonhuman anaimals: theoretical and empirical dissections. *J. Comp. Psychol.* 126 109–113. 10.1037/a0027758 22612372

[B63] Parrill F. (2009). Dual viewpoint gestures. *Gesture* 9 271–289. 10.1075/gest.9.3.01par

[B64] Pika S. Bugnyar T. (2011). The use of referential gestures in ravens (*Corvus corax*) in the wild. *Nat. Commun.* 2 560 10.1038/ncomms1567 PMC4377648 22127056

[B65] Pollick A. S. de Waal F. B. M. (2007). Ape gestures and language evolution. *PNAS* 104 8184–8189. 10.1073/pnas.0702624104 17470779 PMC1876592

[B66] Povinelli D. J. (2000). *Folk Physics for Apes: The Chimpanzee’s Theory of How the World Works.* Oxford: Oxford University Press.

[B67] Reddy V. (2005). “Before the ’third element’: understanding attention to self,” in *Joint Attention: Communication and Other Minds* eds Eilan N. Hoerl C. McCormack T. Roessler J. (New York, NY: Oxford University Press) 85–109. 10.1093/acprof:oso/9780199245635.003.0005

[B68] Rohlfing K. Fritsch J. Wrede B. (2004). “Learning to manipulate objects: a quantitative evaluation of motionese,” in *Proceedings of the Third International Conference on Development and Learning* La Jolla, CA 27.

[B69] Runesson S. (1994). “Perception of biological motion: The KSD-principle and the implications of a distal versus proximal approach,” in *Perceiving Events and Objects* eds Jansson G. Bergström S.-S. Epstein W. (Hillsdale, NJ: Lawrence Erlbaum) 383–405.

[B70] Russon A. Andrews K. (2010). Pantomime in great apes: evidence and implications. *Commun. Integr. Biol.* 4 315–317. 10.4161/cib.4.3/14809 PMC3187895 21980567

[B71] Savage-Rumbaugh S. Shanker S. Taylor T. (1998). *Apes, Language and the Human Mind.* Oxford: Oxford University Press.

[B72] Sterelny K. (2012). *The Evolved Apprentice. How Evolution made Human Unique.* Cambridge MA: The MIT Press 10.7551/mitpress/9780262016797.001.0001

[B73] Strauss S. Ziv M. Stein A. (2002). Teaching as a natural cognition and its relations to preschoolers’ developing theory of mind. *Cogn. Dev.* 17 1473–1487. 10.1016/S0885-2014(02)00128-4

[B74] Studdert-Kennedy M. Goldsmith L. (2003). “Launching language: the gestural origin of discrete infinity,” in *Language Evolution* eds Christiansen M. Kirby S. (Oxford: Oxford University Press) 235–254.

[B75] Tanner J. E. Byrne R. W. (1996). Representation of action through iconic gesture in a captive lowland gorilla. *Curr. Anthropol.* 37 162–173. 10.1086/204484

[B76] Tehrani J. J. Riede F. (2008). Towards an archaeology of pedagogy: learning, teaching and the generation of material culture traditions. *World Archaeol.* 40 316–331. 10.1080/00438240802261267

[B77] Tomasello M. (1999). *The Cultural Origins of Human Cognition.* Cambridge, MA: Harvard University Press.

[B78] Tomasello M. Savage-Rumbaugh S. Kruger A. (1993). Imitative learning of actions on objects by children, chimpanzees, and enculturated chimpanzees. *Child Dev.* 64 1688–1705. 10.2307/1131463 8112113

[B79] Toth N. Schick K. D. Savage-Rumbaugh S. Sevik R. A. Rumbaugh D. M. (1993). Pan the tool-maker: investigations into the stone tool-making and tool-using capabilities of a bonobo (*Pan paniscus*). *J. Archaeol. Sci.* 20 81–91. 10.1006/jasc.1993.1006

[B80] Warglien M. Gärdenfors P. Westera M. (2012). Event structure, conceptual spaces and the semantics of verbs. *Theor. Linguist.* 38 159–193. 10.1515/tl-2012-0010

[B81] Whiten A. (1999). “The evolution of deep social mind in humans,” in *The Descent of Mind: Psychological Perspectives on Hominid Evolution* eds Corballis M. C. Lea S. E. G. (Oxford: Oxford University Press) 173–193.

[B82] Whiten A. Horner V. Marshall-Pescini S. (2005). “Selective imitation in child and chimpanzee: a window on the construal of others’ actions,” in *Perspectives on Imitation: From Neuroscience to Social Science* eds Hurley S. Chater N. (Cambridge, MA: MIT Press) 263–283.

[B83] Whiten A. McGuigan N. Marschall-Pescini S. Hopper L. M. (2009). Emulation, imitation, over-imitation and the scope of culture for child and chimpanzee. *Philos. Trans. R. Soc. B Biol. Sci.* 364 2417–2428. 10.1098/rstb.2009.0069 PMC2865074 19620112

[B84] Woodward J. (2011). “A philosopher looks at tool use and causal understanding,” in *Tool Use and Causal Cognition* eds McCormack T. Hoerl C. Butterfill S. (Oxford: Oxford University Press) 18–50.

[B85] Wynn T. R. Hernandez-Aguilar A. Marchant L. F. McGrew W. C. (2011). ”An ape’s view of the Oldowan” revisited. *Evol. Anthropol.* 20 181–197. 10.1002/evan.20323 22034236

[B86] Zentall T. R. (2001). Imitation in animals: evidence, function and mechanisms. *Cybern. Syst.* 32 53–96. 10.1080/019697201300001812

[B87] Zlatev J. Persson T. Gärdenfors P. (2005a). *Bodily Mimesis as ‘The Missing Link’ in Human Cognitive Evolution.* Lund: Lund University Cognitive Studies 121.

[B88] Zlatev J. Persson T. Gärdenfors P. (2005b). Triadic bodily mimesis is the difference! *Behav. Brain Sci.* 28 720–721. 10.1017/S0140525X05530127

[B89] Zuberbühler K. (2013). Acquired: mirroring and intentional communication in primates. *Lang. Cogn.* 5 133–143. 10.1515/langcog-2013-0008

[B90] Zywiczynski P. Wacewicz S. Sibierska M. (2016). Defining pantomime for language evolution research. *Topoi* 10.1007/s11245-016-9425-9

